# Characteristics Associated with Non-Disclosure of Suicidal Ideation in Adults

**DOI:** 10.3390/ijerph15050943

**Published:** 2018-05-09

**Authors:** Saskia Mérelle, Elise Foppen, Renske Gilissen, Jan Mokkenstorm, Resi Cluitmans, Wouter Van Ballegooijen

**Affiliations:** 1 Research Department, 113 Suicide Prevention, 1100 CE Amsterdam, The Netherlands; r.gilissen@113.nl (R.G.); j.mokkenstorm@113.nl (J.M.); 2 Public Health Service (GGD) Kennemerland, 2015 CK Haarlem, The Netherlands; elisefoppen@gmail.com (E.F.); tlmcluitmans@ggdkennemerland.nl (R.C.); 3 Department of Psychiatry, Amsterdam Public Health Research Institute, VU University Medical Center, 1081 BT Amsterdam, The Netherlands; w.van.ballegooijen@vu.nl; 4 Department of Research & Innovation, GGZ inGeest, Specialized Mental Health Care, 1070 BB Amsterdam, The Netherlands; 5 Section Clinical Psychology, Vrije Universiteit Amsterdam, 1081 BT Amsterdam, The Netherlands

**Keywords:** public health, epidemiology, suicide prevention, adults, suicidal ideation, non-disclosure, risk factors

## Abstract

Suicide prevention efforts often depend on the willingness or ability of people to disclose current suicidal behavior. The aim of this study is to identify characteristics that are associated with non-disclosure of suicidal ideation. Data from the Dutch cross-sectional survey Health Monitor 2016 were used, resulting in 14,322 respondents (age 19+). Multiple logistic regression analyses were conducted to assess the strength of the associations between demographics and health-related characteristics as independent variables, and non-disclosure of suicidal ideation as the dependent variable. The mean age of the respondents was 60 years (SD 16.7) and 45% were male. Of these adults, 5% (*n* = 719) reported suicidal ideation in the past year, nearly half of which (48%) did not disclose suicidal ideation. Non-disclosure was significantly associated with social loneliness (OR = 1.29). Inverse significant associations were found for age (35–49 years, OR = 0.53), poor health status (OR = 0.63), frequent suicidal ideation (OR = 0.48), and severe psychological distress (OR = 0.63). The accuracy of this model was fair (AUC = 0.73). To conclude, non-disclosure is a substantial problem in adults experiencing suicidal ideation. Adults who do not disclose suicidal ideation are more likely to have few social contacts, while they are less likely to experience poor (mental) health and frequent suicidal thoughts.

## 1. Introduction

In the Netherlands, the annual suicide number has increased from 1353 deaths in 2007 to 1894 deaths in 2016 (39% increase). The standardized death rate, however, has remained at the same level since 2013 (11.1 per 100,000 residents) [1]. With regard to risk groups, the suicide rate is markedly highest in males and middle-aged adults; the proportion of males in suicide deaths was 68% compared to 32% females, and 55% were persons aged 40–65 years [2]. Of all the individuals who died by suicide, 62% were not in specialized mental health care [3]. Although effective treatments are available for suicidality, many persons who experience suicidal ideation do not actually seek help. A large-scale cross-national study demonstrated that most people with suicidal ideation or who attempted suicide did not receive treatment [4].

Suicide prevention is targeted at people who are currently experiencing suicidal ideation or who have previously attempted suicide [5]. It is therefore important that people are willing or able to disclose current or previous suicidal behavior [5]. However, attitudinal barriers such as ‘the wish to handle the problem alone’ may hinder the willingness of people to disclose their suicidal ideation [4]. Research has shown that self-concealment in general is related to ruminative thoughts, social disconnection, and a thwarted sense of relatedness [6,7,8,9]. These factors are in turn considered to be risk-factors for suicidal ideation [10].

In recent years, researchers have examined different risk factors of self-concealment (from now on referred to as ‘non-disclosure’). Inhibiting factors of disclosure may result from a dominantly masculine gender role norm—the notion that talking about difficult feelings would lead to a loss of masculine power [11]. Accordingly, males showed a higher tendency toward non-disclosure of difficult feelings [12]. Secondly, a lack of available confidants was also stated as a reason for non-disclosure of suicidal ideation [13]. This lack of availability, i.e., social loneliness, and the absence of an intimate relationship or a close emotional attachment, i.e., emotional loneliness, have been found to be related to non-disclosure of personal distress [12]. Thirdly, high levels of distress have been found to be positively related to non-disclosure of emotional events [14]. Furthermore, non-disclosure has been linked to poorer physical well-being, while disclosure of personal information, such as secrets, has been related to positive health benefits [5,15]. Finally, higher rates of alcohol consumption were associated with a higher chance of non-disclosure [16].

In 2017, the Dutch National Suicide Prevention Center started an implementation program in six pilot regions of the Netherlands [17]. The main aims of these Suicide Prevention Action NETwork (SUPRANET) communities are to encourage the general public to talk about suicide, to break the taboo, and to improve the care of high-risk groups [17]. By doing so, insight into specific risk factors of non-disclosure of suicidal ideation might enable health professionals and gatekeepers to reach these individuals experiencing suicidal ideation and direct them to the available professional help.

However, to date, no studies have assessed which factors are related to non-disclosure of suicidal ideation in the general population, while empirical evidence about the prevalence is also scarce. Knowledge and understanding of this subject are important as the disclosure of suicidal ideation is often seen as a crucial first step in the help-seeking process [18]. Therefore, the current study aims to gain insight into factors that are associated with non-disclosure of suicidal ideation. Based on previous research, it is expected that non-disclosure of suicidal ideation is associated with male gender, poor health status, severe psychological distress, emotional and social loneliness, and excessive drinking.

## 2. Materials and Methods

### 2.1. Study Design and Population

This study was based on a secondary analysis of data derived from the ‘Health Monitor Adults and Elderly 2016 study’ that was carried out by Public Health Service Kennemerland, in the northwest Netherlands [19]. This ‘Health Monitor’ was a large-scale survey and contained up-to-date information about physical and mental health, chronic diseases, lifestyle behaviors, social functioning, and the environment.

All public health services in the Netherlands participated in this research between September and December 2016 at the behest of the National Institute for Public Health and the Environment (RIVM) and in collaboration with Statistics Netherlands (CBS) [19,20]. With these results, municipal executives and health policy makers were informed about the health status and well-being of inhabitants and could organize regional and local activities for health promotion. Public Health Service Kennemerland added some extra themes to this national survey, including suicidal ideation, within the context of being part of the Dutch SUPRANET communities [17].

Statistics Netherlands randomly sampled participants from municipality registries stratified by age group (19–64 years and 65+). Individuals were excluded if they lived in nursing homes or mental health institutions, or if they were imprisoned or were staying in accommodations for asylum seekers [19,20]. For each age group, the sample size was calculated based on a formula which combined the size of the total target population and the expected response rate to provide sufficient statistical power [20]. In total, 40,297 adults in Kennemerland were approached (27,023 19–64 years; 13,274 65+ years) and 15,600 participated, leading to an overall response rate of 39% (8319 participants 19–64 years, 31% response rate; 7281 participants 65+ years, 55% response rate).

### 2.2. Procedure

Data were collected from September to December 2016. Selected individuals were invited by postal invitation to participate in an online survey and received a personal log-on code. For those over 75 years of age, a paper questionnaire was included. The invitation also informed participants about the enrichment of the data by linking the survey dataset to other data from Statistics Netherlands, i.e., income, ethnicity, year of birth, and gender [19,20].

Those who did not reply within three weeks received their log-on codes for the online survey as well as a paper-based version of the questionnaire. A second reminder was sent five weeks after the first reminder and included log-on codes for those less than 75 years of age; those above 75 years also received a written questionnaire. Due to a low response rate in the youngest age group, those aged between 19 to 34 years received a post card as third reminder [19].

In accordance with the Dutch Personal Data Protection Act, privacy of the respondents was warranted by separating data that was used to communicate with respondents from the research dataset [21]. In addition, personal information was never visible in the dataset, information was only used for statistical purposes and no other institutions could acquire access to the data without permission from a national registration committee [21]. Furthermore, by submitting the questionnaire, respondents authorized their data for further processing. The Medical Ethics Committee of the Academic Medical Center in Amsterdam reviewed the study protocol and deemed that the national ‘Health Monitor Adults and Elderly 2016’ did not fall under the Medical Research Involving Subjects Act (W16_166 #16.196) [20].

### 2.3. Measurements

#### 2.3.1. Suicidal Ideation and Non-Disclosure

Suicidal ideation was assessed by a single item that is commonly used in Dutch public health research [22]. This question was carefully developed based on subject-matter knowledge, but has not been tested for validity. Suicidal ideation was quantified by asking adults the following question: ‘Have you seriously thought about ending your life in the past 12 months?’. Response options ranged from ‘no’ (1) to ‘very often’ (5) and ‘I do not want to answer this question’ (6). Scores were dichotomized into ‘suicidal ideation’ (scores 2–5) and ‘no suicidal ideation’ (score 1) for descriptive purposes. Respondents who were not willing to answer this question were declared as missing values. Suicidal ideation scores were also used as an independent continuous variable in the logistic regression analyses to measure frequency of suicidal ideation. Suicidal ideations scores were recoded for this purpose into scores ranging from ‘a few times’ (1) to ‘very often’ (4). 

The dependent variable non-disclosure of suicidal ideation was measured in those who gave an affirmative answer to the previous question. To measure non-disclosure, the researchers used a question that has been developed for the implementation research of the Dutch SUPRANET communities by Public Health Service Fryslân [17]: ‘Did you discuss your suicidal thoughts with one or more others?’ and ‘Who did you talk to?’. For this study, the answers to the first question were used, and ‘yes’ and ‘no’ were recoded into ‘non-disclosure of suicidal ideation’ (1 = answer no) and ‘disclosure of suicidal ideation’ (0 = answer yes).

#### 2.3.2. Demographics

Information on gender was assessed by a question in the survey, and missing data on gender were completed by data from Statistics Netherlands. Age was used as a continuous variable for descriptive purposes and categorized into five categories, i.e., ‘19–34’, ‘35–49’, ‘50–64’, ‘65–74’, ‘75+’ years, for both descriptive statistics and the logistic regression analyses (see Section 2.4 Statistical analyses). Educational level was used for descriptive purposes and was based on self-reported answers about the highest completed qualification. Accordingly, answers were categorized into three educational levels (see Table 1). Respondents were further asked about their marital status. Five response options were recoded into four categories: ‘married/cohabiting’ (0), ‘unmarried’ (1), ‘divorced’ (2), and ‘widowed’ (3).

#### 2.3.3. Perceived Health Status

General self-rated health was measured in a single-item question on a 5-point Likert scale, which showed adequate predictive validity for mortality rate [23] . Respondents were asked: ‘How do you rate your health in general?’ with answers ranging from ‘very good’ (1) to ‘very bad’ (5). This score was used as a continuous variable.

#### 2.3.4. Psychological Distress

Psychological distress was assessed by the Dutch version of the 10-item Kessler Psychological Distress Scale (K10). The K10 is a brief symptom scale that has been developed for epidemiological research and focuses on anxiety and depression [24]. The Dutch version has adequate convergent validity and criterion validity relative to measures of depression among a community sample [25]. Respondents were asked to rate each symptom on a 5-point Likert scale, ranging from ‘always’ (5) to ‘never’ (1). All questions referred to the past month. Respondents who had at least 3 missing items were excluded from the analyses. The overall sample mean was used to replace 1 or 2 missing values [22,26]. The internal consistency in our study sample was checked and approved (Cronbach’s α = 0.91). Total scores were computed by adding up the item scores. Based on cut-off values in public health, adults were divided into ‘mild’ (10–15), ‘moderate’ (16–29), and ‘severe’ (30–50) distress [22,26].

#### 2.3.5. Social and Emotional Loneliness

Loneliness was assessed using the De Jong Gierveld loneliness scale. This scale is based on two dimensions, i.e., social and emotional loneliness; emotional loneliness refers to ‘the absence of an intimate relationship or a close emotional attachment’, and social loneliness to ‘the absence of a broader group of contacts or an engaging social network’ [27]. Response options for the 11 statements included ‘yes’ (1), ‘more or less’ (0), and ‘no’ (0). Five positively formulated items were reverse scored. Total scores for social (5 items) and emotional loneliness (6 items) were computed, provided that there were no missing values on each subscale. Both total scores were used as continuous variables. The internal consistency was good in our study sample (Cronbach’s α = 0.80 and α = 0.85 for social and emotional loneliness, respectively).

#### 2.3.6. Excessive Drinking

In this study, excessive drinking was defined as consuming at least 21 alcohol units per week for males and at least 14 units per week for females [22,28]. Alcohol consumption was measured with the quantity, frequency, and variability method [22,28]. Respondents were asked to indicate their average number of drinking days, separately for per week and weekend, as well as the average number of alcohol units consumed during a drinking day. Response options ranged from ‘four weekdays’/‘three weekend days’ to ‘less than one day’ or ‘I never drink during week/weekend days’. The number of alcohol units ranged from ‘one glass’ to ‘11 glasses or more’. Scores ‘7–10’ and ‘11 or more’ were recoded into 8.5 and 15, respectively. Total alcohol units per week were calculated by multiplying the average of drinking days (0.5–7) and the average of alcohol units (1–15). Total scores were dichotomized into ‘excessive drinking’ (≥21 M/≥14 F) and ‘no excessive drinking’ groups (<21 M/<14 F).

### 2.4. Statistical Analyses

First, descriptive analyses were used to calculate the (unweighted) prevalence of suicidal ideation and non-disclosure and to examine the distribution of the different characteristics among the study population (see Table 1). Next, in order to find factors that are associated with non-disclosure of suicidal ideation, multiple logistic regression analyses were conducted (see Table 2). The assumptions of the logistic regression analyses (linearity of the relationship between the independent variable and outcome variable, and multicollinearity) were tested. In the case of non-linear relationships, dummy variables based on public health categories were used in the analyses instead of the continuous variable, i.e., age, psychological distress and excessive drinking [26,28]. The same procedure was followed as earlier described by Boerema et al. (2016) [26]. First, a sequence of bivariate analyses was performed to assess the Odds Ratio (OR) of each factor separately. Secondly, all factors were included together in a multivariate model (‘basic model’). Third, a backwards selection procedure was done to form a final model that included the strongest factors related to non-disclosure of suicidal ideation (*p* < 0.05) [29]. Further, the effect sizes of the final factors were determined and an OR ≥ 2 (or ≤0.5) was considered as practically relevant [30]. Lastly, the accuracy of the final model was classified by using the statistical value for the area under the curve (AUC) of the ROC-curve: excellent (0.9–1.0), good (0.8–0.9), fair (0.7–0.8), poor (0.6–0.7), and fail (0.5–0.6) [31].

All analyses were performed using SPSS 23.0 and we maintained two-tailed 95% confidence intervals for significance testing.

## 3. Results

### 3.1. Study Population

Table 1 presents demographics and health-related characteristics of the study population (*N* = 14,322), stratified by respondents’ suicidal ideation and disclosure of suicidal ideation.

#### Characteristics of Respondents

In total, 14,620 of the 15,600 participants answered the question about suicidal ideation at the end of the questionnaire, and 2% (*n* = 298) reported that they did not want to answer this question. The remaining respondents (*n* = 14,322) were largely elderly adults, on average 60 years old (SD = 16.7), and the youngest adults were in a minority (10% 19–34 years). The proportion of males was 45%, and educational levels were, more or less, evenly represented. Seven out of ten respondents were married or cohabiting. Table 1 shows further information on sample characteristics.

Of the responding adults, 5% (*n* = 719) reported suicidal ideation in the past year. The frequency of suicidal ideation was as follows. 57% (*n* = 412) reported having had suicidal ideation ‘a few times’, 31% (*n* = 220) reported ‘sometimes’, and 12% (*n* = 87) ‘(very) often’. In addition, nearly half of the respondents with suicidal ideation (48%, *n* = 348) did not disclose their suicidal thoughts, as opposed to the 52% that reported disclosure.

### 3.2. Characteristics Associated with Non-Disclosure of Suicidal Ideation in Adults

Table 2 shows the results of the logistic regression analyses used to assess the strength of associations between demographics and health-related factors as independent variables, and non-disclosure of suicidal ideation as dependent variables.

#### 3.2.1. Basic Multivariate Model

Firstly, the bivariate analyses showed that of all demographical factors, only age was significantly associated with non-disclosure of suicidal ideation (see Table 2, bivariate). Regarding health-related factors, perceived health status, suicidal ideation, psychological distress, and social loneliness were significantly related to non-disclosure.

The basic model, including all potential factors, revealed that the age group ‘65–74 years’ compared to ‘19–34 years’ had an increased likelihood of non-disclosure. Furthermore, poor health status and frequent suicidal ideation were inversely related to non-disclosure, indicating that they decreased the likelihood of non-disclosure. In addition, a higher level of social loneliness increased the likelihood of non-disclosure (see Table 2, basic model).

#### 3.2.2. Final Multivariate Model

Using a backwards elimination procedure, the final model demonstrated the following factors to be most strongly related to non-disclosure of suicidal ideation (see Table 2). Age (35–49 years, OR = 0.53, 95% CI = 0.34–0.81), poor health status (OR = 0.63, 95% CI = 0.51–0.78), frequent suicidal ideation (OR = 0.48, 95% CI = 0.37–0.62), severe psychological distress (OR = 0.63, 95% CI = 0.42–0.94), and high level of social loneliness (OR = 1.29, 95% CI = 1.17–1.41). Although the variable age ‘65–74 years’ was included in the final model based on the backwards selection procedure, it was no longer significantly associated with non-disclosure.

Only the factor ‘frequent suicidal ideation’ was classified as practically relevant (OR ≤ 0.5). The accuracy of the final model was fair based on an AUC of 0.73 (95% CI = 0.69–0.77), *p* < 0.001.

## 4. Discussion

The current study aimed to identify which demographics and health-related characteristics are associated with non-disclosure of suicidal ideation. Our main findings are discussed successively.

### 4.1. Interpretation of Key Findings

First, the results showed that 48% of the adults who have suicidal ideation do not disclose their suicidal thoughts to others. This finding is important in that it indicates non-disclosure is a substantial problem in suicidal adults. Albeit from a different study population, the results are in line with previous research. One retrospective study found that 27% of adults who died by suicide had not talked about their suicidal thoughts with a confidant, while 8% had denied having suicidal thoughts when questioned by their physician [6]. In another study, 50% of senior adults (60+) had not disclosed their current suicidal thoughts to a health care provider during treatment [32]. These findings imply that a substantial group of adults who have suicidal ideation remain invisible for suicide prevention efforts.

Secondly, the findings of this study revealed that adults who do not disclose their suicidal ideation are more likely to have few social contacts, while they are less likely to be in their thirties or forties and less likely to experience poor health, frequent suicidal ideation, and severe psychological distress. Of these final factors, poor health status, frequent suicidal ideation, and social loneliness were most strongly related to non-disclosure (*p* < 0.001), whereby the factor suicidal ideation reached practical relevance. The accuracy of this final model was satisfying indicating an acceptable model fit.

The empirical findings presented here fit with previous research, in which a lack of confidants was mentioned as one of the main reasons for non-disclosure of suicidal ideation [13]. In contrast to previous research, no significant association was found between emotional loneliness and non-disclosure of suicidal ideation [12]. Although we cannot draw any conclusions about causality, these findings suggest that a lack of availability of social support might withhold people from sharing their suicidal thoughts. Besides, we did not find any significant relationship between marital status and the non-disclosure of suicidal ideation. This might further support the idea that the availability of social support seems more important for disclosure of suicidal ideation than the intimacy of a relationship.

Gender differences were not present in this study, indicating that male gender is not a demographical risk factor for non-disclosure of suicidal ideation. Secondary analyses showed that frequent suicidal ideation, i.e., having had suicidal thoughts (very) often, was 12% in both males and females. Given these facts, it is promising that the current study suggests that that the likelihood that males disclose their suicidal thoughts seems to be equal to females. Unlike gender, age appears to be a relevant demographical factor for disclosure of suicidal ideation. The bivariate and multivariate analyses indicated that the age group 65–74 years was at risk for non-disclosure. This is worrisome, since suicide rates have recently increased among Dutch senior adults (60+) [2]. In addition, adults in their thirties or forties were more likely to disclose their suicidal ideation.

Contrary to our expectations, the results showed that poor health status and severe psychological distress were positively related to disclosure of suicidal ideation. These findings contradict previous research in which a high level of depressive symptoms was negatively related to disclosure tendencies [14]. Other research, however, indicated that ‘poor quality of life’ and ‘a prior history of specialized mental health care’ increased the likelihood of disclosure of suicidal ideation to a health care provider [32]. The unexpected findings could therefore be due to the fact that adults with severe (mental) health complaints have a higher chance of receiving specialized mental health care treatment, which in turn, increases the likelihood of disclosure. Lastly, excessive drinking was not significantly related to non-disclosure of suicidal ideation. A recent study found that male adults, who used alcohol to cope with negative effects, were more vulnerable to suicidal behavior [33]. Therefore, drink-related coping motives might mediate the relationship between alcohol use and non-disclosure. This understudied area needs further research to replicate and understand the relationships.

### 4.2. Strengths and Limitations

An important strength of the present study is the use of a large-scale sample of adults from the general population. These adults completed commonly used public health questionnaires about their health status and wellbeing. Consequently, this study contributes to the field of suicide prevention research by creating a clear view of characteristics of adults who have suicidal ideation and do not talk about their suicidal thoughts.

The use of data from the ‘Health Monitor 2016’ led to some limitations. First of all, the use of a cross-sectional approach means that causal relationships cannot be determined. Secondly, the reliance on self-reported data might be biased to fit in with socially acceptable norms. However, 92% of the respondents answered the delicate question about frequency of suicidal ideation, and the prevalence estimate coincides with previous research [34]. It is noteworthy to mention that 2% (*n* = 298) did not want to answer the question related to suicidal ideation. This subgroup might also reflect non-disclosure of suicidal ideation, and leaving out this specific group is another study limitation. Thirdly, adults 65 years and older were overrepresented in the study (47% of the study group versus 24% in the Netherlands), as a consequence of the higher response rate in this age group. The overrepresentation of older adults may affect the generalizability of our results. Public Health Service Kennemerland has used weighted prevalence estimates to inform local health policy makers, yielding a somewhat lower percentage of non-disclosure (43%) [19]. This is possibly due to correction for the underrepresentation of younger adults.

### 4.3. Practical Implications

The current study makes some important contributions to practice. Firstly, community gate keepers are trained to identify suicide risk, to make contact and ‘open the gates’ for help-seeking [17]. A recent observational study demonstrated significant improvements in gatekeepers’ knowledge of suicide prevention and their confidence to discuss suicidality after gatekeeper training [35]. Gatekeeper training could therefore make gatekeepers aware of risk-groups for non-disclosure. In this case, gatekeepers are challenged to identify adults who are socially isolated and sometimes have suicidal thoughts. Opportunities for these risk groups could be offered, for example, in the socioeconomic sector by employees who encounter socially vulnerable people in their daily work (e.g., vocational experts, bank employees, insurance doctors, and debt counselors).

Secondly, this study implies that adults who sometimes have suicidal ideation and experience fair (mental) health are in a ‘window of opportunity to change’. Preventing high suicide risk from occurring might be critical for members of this group, while for others, suicidal ideation might resolve with no intervention. A review suggested that in both adolescent and adult populations, it seems important for professionals to ask about suicidal ideation and talk about these thoughts [36]. Clinical experience has shown that patients may feel relieved when talking about their suicidal thoughts. They may also feel better understood and less lonely; talking may also help them to structure thoughts [37]. Assuming that talking is beneficial, direct and personal forms of influence are needed to reach those individuals experiencing suicidal ideation in need of help [18]. For instance, the intervention ‘Life Goals’ aims to increase the participation of people who are socially vulnerable by means of sporting activities, and may encourage participants to openly discuss suicidal thoughts [38].

## 5. Conclusions

This study has shown that a substantial group of adults who have suicidal ideation remain invisible for suicide prevention efforts. Adults who do not disclose their suicidal ideation are more likely to have few social contacts, while they are less likely to experience poor (mental) health and frequent suicidal ideation. Suicide prevention programs could train gatekeepers to reach these ‘invisible’ individuals experiencing suicidal ideation to openly discuss suicidal thoughts and direct those in need to the available professional help. Lastly, given the paucity of research on this topic, Dutch SUPRANET communities can replicate this study design to identify (new) risk factors of non-disclosure of suicidal ideation.

## Figures and Tables

**Table 1:** Characteristics of the study population.

Characteristics	No Suicidal Ideation Past Year (*N* = 13,603)	Suicidal Ideation Past Year (*N* = 719)	Total (*N* = 14,322)	Disclosure of Suicidal Ideation (*N* = 363)	Non-Disclosure of Suicidal Ideation (*N* = 348)
Demographics	*n* = 13,603	*n* = 719	*n* = 14,322	*n* = 363	*n* = 348
Age, mean (SD)	60.3 (16.6)	56.3 (18.4)	60.0 (16.7)	55.4 (19.0)	56.7 (17.7)
Age category (%)					
19–34 years	1274 (9.4)	113 (15.7)	1387 (9.7)	59 (16.3)	54 (15.5)
35–49 years	2174 (16.0)	133 (18.5)	2307 (16.1)	84 (23.1)	49 (14.1)
50–64 years	3645 (26.8)	214 (29.8)	3859 (26.9)	103 (28.4)	109 (31.3)
65–74 years	3774 (27.7)	134 (18.6)	3908 (27.3)	52 (14.3)	81 (23.3)
75+	2736 (20.1)	125 (17.4)	2861 (20.0)	65 (17.9)	55 (15.8)
Gender (%)	*n* = 13,603	*n* = 719	*n* = 14,322	*n* = 363	*n* = 348
male	6115 (45.0)	332 (46.2)	6447 (45.0)	163 (44.9)	167 (48.0)
female	7488 (55.0)	387 (53.8)	7875 (55.0)	200 (55.1)	181 (52.0)
Educational level (%)	*n* = 13,497	*n* = 710	*n* = 14,207	*n* = 358	*n* = 345
low (primary, lower vocational)	4821 (35.7)	242 (34.1)	5063 (35.6)	126 (35.2)	114 (33.0)
medium (secondary, secondary vocational)	4341 (32.2)	269 (37.9)	4610 (32.4)	134 (37.4)	132 (38.3)
high (≥bachelor’s degree)	4335 (32.1)	199 (28.0)	4534 (31.9)	98 (27.4)	99 (28.7)
Marital status (%)	*n* = 13,474	*n* = 707	*n* = 14,181	*n* = 358	*n* = 341
married/cohabiting	9647 (71.6)	341 (48.2)	9988 (70.4)	181 (50.6)	158 (46.3)
unmarried	1294 (9.6)	152 (21.5)	1446 (10.2)	78 (21.8)	73 (21.4)
divorced	1035 (7.7)	115 (16.3)	1150 (8.1)	57 (15.9)	56 (16.4)
widowed	1498 (11.1)	99 (14.0)	1597 (11.3)	42 (11.7)	54 (15.8)
Health-related factors					
Perceived health, mean (SD)	*n* = 13,529	*n* = 712	*n* = 14,241	*n* = 360	*n* = 344
very good-poor health status (1–5)	2.08 (0.72)	2.80 (0.89)	2.11 (0.74)	2.97 (.94)	2.62 (0.80)
Psychological distress (%)	*n* = 13,536	*n* = 713	*n* = 14,249	*n* = 362	*n* = 343
mild	8733 (64.5)	77 (10.8)	8810 (61.8)	31 (8.6)	46 (13.4)
moderate	4517 (33.4)	397 (55.7)	4914 (34.5)	174 (48.1)	219 (63.8)
severe	286 (2.1)	239 (33.5)	525 (3.7)	157 (43.4)	78 (22.7)
Emotional Loneliness, mean (SD)	*n* = 13,230	*n* = 693	*n* = 13,923	*n* = 353	*n* = 335
no-severe loneliness (0–6)	0.99 (1.6)	3.24 (2.3)	1.11 (1.8)	3.26 (2.3)	3.18 (2.3)
Social Loneliness, mean (SD)	*n* = 13,301	*n* = 703	*n* = 14,004	*n* = 357	*n* = 341
no-severe loneliness (0–5)	1.53 (1.7)	3.10 (1.9)	1.61 (1.7)	2.88 (1.9)	3.31 (1.9)
Excessive drinking (%)	*n* = 13,105	*n* = 687	*n* = 13,792	*n* = 345	*n* = 339
≥21/14 units/week	1259 (9.6)	83 (12.1)	1342 (9.7)	45 (13.0)	38 (11.2)
<21/14 units/week	11,846 (90.4)	604 (87.9)	12,450 (90.3)	300 (87.0)	301 (88.8)

Note: (SD) = standardized deviation. Note: Rounding the sum of the percentage in each category can be somewhat higher or lower than 100%, for example 99.9% or 100.1%. Note: The number of respondents per characteristic (variable) varies depending on the number of missing values. For each characteristic the number of respondents per subgroup adds up to a total group. For example the variable gender within the group of respondents with ‘no suicidal ideation past year’: 6115 males + 7488 females = 13,603 respondents.

**Table 2:** Characteristics associated with non-disclosure of suicidal ideation in adults, and results of the backwards selection procedure.

Non-Disclosure of Suicidal Ideation in Adults—Yes or No (Suicidal Ideation = 719, Non-Disclosure, *n* = 348)						
Characteristics	Multivariate					
	Bivariate ^1^		Basic Model ^2^		Final Model ^3^	
	OR	95% CI	OR	95% CI	OR	95% CI
Demographics						
Age						
19–34 years	1.00		1.00			
35–49 years	0.64	0.38–1.06	0.65	0.36–1.16	0.53	0.34–81 **
50–64 years	1.16	0.73–1.83	1.47	0.81–2.68		
65–74 years	1.70	1.03–2.83 *	2.04	1.03–4.02 *	1.46	0.95–2.26
75 +	0.93	0.55–1.55	1.09	0.50–2.37		
Gender						
female	1.00		1.00			
male	1.13	0.84–1.52	1.20	0.84–1.70		
Educational level						
low	0.90	0.61–1.31	1.03	0.65–1.63		
medium	0.98	0.68–1.41	1.19	0.78–1.82		
high	1.00		1.00			
Marital status						
married/cohabiting	1.00		1.00			
unmarried	1.07	0.73–1.57	1.54	0.92–2.56		
divorced	1.13	0.74–1.72	1.43	0.85–2.43		
widowed	1.47	0.93–2.32	1.49	0.76–2.93		
Health-related factors						
Health status very good-poor health status(1–5)	0.63	0.52–0.75 ***	0.60	0.47–0.76 ***	0.63	0.51–0.78 ***
Suicidal ideation a few times-very often (1–4)	0.46	0.37–0.57 ***	0.45	0.34–0.59 ***	0.48	0.37–0.62 ***
Psychological distress						
mild (10–15)	1.00		1.00			
moderate (16–29)	0.85	0.52–1.39	1.01	0.57–1.79		
severe (30–50)	0.34	0.20–0.57 ***	0.69	0.34–1.39	0.63	0.42–0.94 *
Emotional Loneliness no-severe loneliness (0–6)	0.99	0.92–1.05	1.01	0.91–1.12		
Social Loneliness no-severe loneliness (0–5)	1.13	1.05–1.23 **	1.25	1.11–1.42 ***	1.29	1.17–1.41 ***
Excessive drinking						
<21/14 units/week	1.00		1.00			
≥21/14 units/week	0.84	0.53–1.33	0.72	0.42–1.22		

*p* < 0.05 *, *p* < 0.01 **, *p* < 0.001 ***; ^1^ binary logistic regression; ^2^ multiple logistic regression; ^3^ final model including only the strongest factors using a backwards selection procedure.
